# Augmenting brain function with meditation: can detachment coincide with empathy?

**DOI:** 10.3389/fnsys.2015.00141

**Published:** 2015-10-08

**Authors:** Shirley Telles, Nilkamal Singh, Acharya Balkrishna

**Affiliations:** Patanjali Research FoundationHaridwar, India

**Keywords:** meditation, detachment, empathy, dmPFC, vmPFC

## Background and aim

Meditation practices aim at modifying the emotions by reducing reactivity to both pleasant and unpleasant emotions (Sperduti et al., 2012; Reva et al., 2014). Meditation also regulates the attention by reducing distractibility and directing focused attention to the object of meditation (Sperduti et al., 2012). In many meditation techniques attention is directed to ongoing experiences without the intention to analyze, judge, get involved, or expect anything which can lead to detachment (Tei et al., 2009). Detachment here was taken to mean reducing or removing the need to control, possess, and be influenced by the actions of people or circumstances. Detachment or remaining dispassionate is ideally combined with empathy. The aim of the present article is to discuss the neurophysiological basis by which detachment and empathy may co-exist in meditation.

## Review of relevant articles

Tei et al. (2009) found differences between Qigong meditators and controls in the resting state using standardized low resolution electromagnetic tomography (sLORETA; *t*-tests for unpaired data, Tei et al., 2009). The Qigong meditation includes practices for self-regulation of the body and mind (Pan et al., 1994). In the study by Tei et al. (2009) differences between groups were seen in the delta EEG band. In meditators brain areas involved in detecting and integrating internal and external sensory information were activated, whereas appraisal systems were inhibited. In non-meditators film generated emotion which is emotion in response to external stimuli was associated with activity in the occipitotemporoparietal cortex, lateral cerebellum, hypothalamus, and regions involving the anterior temporal cortex, amygdala, and hippocampal formation; whereas recall generated emotion, associated with the response to interoceptive sensory stimuli was associated with activation in the anterior insular cortex (Reiman et al., 1997).

A quantitative meta-analysis of 10 neuroimaging studies with 91 meditators, tested the hypothesis that a common neural network exists for different meditations since all meditations aim at inducing relaxation, regulating attention, and developing detachment from one's thoughts (Sperduti et al., 2012). An activation likelihood estimation (ALE) of the 10 fMRI studies showed activation during meditation in the left caudate nucleus (body of the caudate), entorhinal cortex and medial prefrontal cortex. This suggests that a common neural network may exist for meditation techniques which have comparable regulation of attention and goals. Changes in activity in the medial prefrontal cortex (MPFC) can be associated with mental activity during introspection (Gusnard et al., 2001). The ventral MPFC shows task-induced decreases in activity even when the task consists, for example, of lying quietly (Shulman et al., 1997). Hence, the ventral MPFC is believed to be engaged in actively maintaining a default state, which gets attenuated even by passive activities (e.g., lying quietly with eyes closed or passively viewing a stimulus; Raichle et al., 2001). In addition as detailed below the ventral MPFC, along with the temporo-parietal junction and medial temporal lobe are involved in cognitive aspects of empathy (Shamay-Tsoory, 2011). In contrast to the ventral MPFC, changes in the dorsal MPFC include both increases and decreases in activity (Pardo et al., 1993; Shulman et al., 1997). Increased activity was found in BA 8, 9, and 10 and the adjacent paracingulate sulcus of the dorsal MPFC (Castelli et al., 2000). Gusnard and others showed increased activity in the dorsal MPFC during mental activity associated with introspection (Gusnard et al., 2001).

Mental activity associated with introspection is possibly part of a continuum in the mental state induced by meditation which could lead to detachment (Atchley, 1997).

This is supported by a sLORETA study of experienced meditators practicing five types of techniques, viz., Tibetan-Buddhist, Qigong, Sahaja-Yoga, Ananda Marga Yoga, and Zen (Lehmann et al., 2012). Eight EEG frequency bands (delta through gamma) were computed for 171 functional connectivities between regions (*t*-tests for unpaired data), and showed “lagged coherence.” All significant differences between meditation and before/after states showed lower coherence during meditation for the five meditations and eight EEG bands. This suggested that functional interdependence between brain regions was globally reduced. The reduced functional interdependence was considered to be related to minimized interaction and constraints on the self process (which includes self-awareness and embodiment), leading to non-involvement and detachment (Lehmann et al., 2012). Detachment can help an individual to get mental peace (Sonnetag et al., 2010). However, in order to contribute usefully to society empathy and compassion are essential. Empathy and compassion are seemingly similar social emotions, however they alter brain activation in distinct, non-overlapping neural networks, and change affective responses with opposite valence (Klimecki et al., 2014).

Empathy is the ability to understand other's feelings (Bird et al., 2010). However, when faced with the suffering of others, intense sharing of the pain experiences by others can cause empathic distress, and reduce helping behavior (Batson et al., 1987; Eisenberg et al., 1989). It was speculated by Klimecki et al. (2014) that compassion could be a potential remedy. Compassion has been defined as a feeling of concern for the suffering of others, associated with the motivation to help (Keltner and Goetz, 2007). Klimecki et al. (2014) demonstrated that after empathy training viewing videos depicting suffering resulted in negative affect and brain activation in regions previously associated with empathy for pain. The areas were the anterior insula, and anterior mid cingulate cortex (Fan et al., 2011; Lamm et al., 2011). When the same individuals (Klimecki et al., 2014) were subsequently given training in compassion and asked to view videos depicting comparable suffering, the negative affect was reversed and brain activations occurred in non-overlapping areas including the ventral striatum, pregenual anterior cingulate cortex, and medial orbito frontal cortex. Klimecki et al. (2014) concluded that compassion training could actually reduce empathic distress and increase emotional resilience. Compassion is an inherent part of a meditation technique called compassion-meditation (CM), which is closely related to two other widely studied meditation techniques, that is, mindfulness-based meditation (MM) and Loving-Kindness meditation (LKM; Hofmann et al., 2011).

There is evidence which supports two separate systems for cognitive and for emotional aspects of empathy apart from the areas involved in empathy training described earlier (Klimecki et al., 2014). The areas involved in cognitive empathy, are particularly relevant to this article. They are the ventral MPFC, temporo-parietal junction, and medial temporal lobe (Shamay-Tsoory, 2011). As discussed above the dorsal MPFC is associated with detachment (Gusnard et al., 2001). Hence, the MPFC in addition to other functions, is concerned with both empathy (the ventral MPFC) and with detachment (the dorsal MPFC).

Brain activity using fMRI was assessed while novice and experienced meditators practiced loving-kindness-compassion meditation (LKCM; Lutz et al., 2008). Affective reactivity was probed by presenting emotional and neutral sounds during meditation and control periods. When emotional sounds were presented there was activation in the insula and cingulate cortices during meditation compared to rest (ANOVA; paired *t*-tests). Also, during meditation (compared to rest), in response to emotional and neutral sounds, there was increased activation in the amygdala, right temporo-parietal junction, and right posterior superior temporal sulcus. The results support the speculation that LKCM activates brain circuits related to empathy in response to emotional stimuli.

The studies cited above suggest that LKM causes functional changes in specific areas and in inter-connections between areas, to generate social consciousness and empathy. However, as the names suggest, LKM and CM emphasize loving kindness and compassion, respectively, in addition to empathy.

A study was designed to examine the effects of focused-attention meditation (FAM) and loving-kindness meditation (LKM) during the performance of cognitive and affective tasks (Lee et al., 2012). There were 22 practitioners of FAM (11 experts and 11 novices) and similarly for LKM, 11 experts and 11 novices. Comparisons were between states (meditation vs. baseline) and expertise (experts vs. novices; paired and unpaired *t*-tests). A conjunction approach was used to reveal regions common to the expert meditation state. Both FAM and LKM appeared to influence the neural responses to affective pictures.

The difference between FAM and LKM was apparent when participants were given emotion-processing tasks, involving viewing affective pictures. When viewing sad faces the FAM practitioners showed activation in areas related to attention whereas in the LKM practitioners changes occurred in areas associated with compassion and emotional regulation.

## Summary

These findings showed that different meditations are associated with distinct neural activity during sustained attention and processing emotions. Hence, meditation causes changes in parts of the brain and modifies brain activity, in distinct ways based on the meditation technique.

To our knowledge no study has checked whether detachment and empathy can co-exist as a result of meditation; and the pathway has not been determined. The dorsal MPFC showed increased activation associated with mental activity during introspection (Gusnard et al., 2001), which is believed to lead to detachment (Raichle et al., 2001). The ventral MPFC has been associated with cognitive empathy (Shamay-Tsoory, 2011). The ventral and dorsal MPFC are functionally connected via the amygdala (Kim et al., 2011). It is speculated that this neural circuit, involving the dorsal and ventral MPFC and the amygdala, a circuit which could be the basis for simultaneous detachment and empathy. It hence appears that detachment and empathy are associated with activation in closely connected areas of the brain.

## Recommendations for future studies

The above discussion suggests that detachment and empathy can be experienced simultaneously through meditation. A study of the effects of meditation would be interesting to test whether detachment and empathy can indeed co-exist. It would also be of interest to understand whether following the necessary training empathy could lead to compassion. The present speculation suggests that through meditation an individual can reach a mental state which is disengaged and detached, but retains the choice to engage with specific situations based on empathy.

### Conflict of interest statement

The authors declare that the research was conducted in the absence of any commercial or financial relationships that could be construed as a potential conflict of interest.

## References

[B1] Atchley R. C. (1997). Everyday mysticism: spiritual development in later adulthood. J. Adult Dev. 4, 123–134.

[B2] Batson C. D. Fultz J. Schoenrade P. A. (1987). Distress and empathy: two qualitatively distinct vicarious emotions with different motivational consequences. J. Pers. 55, 19–39. 3572705 10.1111/j.1467-6494.1987.tb00426.x

[B3] Bird G. Silani G. Brindley R. White S. Frith U. Singer T. (2010). Empathic brain responses in insula are modulated by levels of alexithymia but not autism. Brain 133, 1515–1525. 10.1093/brain/awq060 20371509 PMC2859151

[B4] Castelli F. Happé F. Frith U. Frith C. (2000). Movement and mind: a functional imaging study of perception and interpretation of complex intentional movement patterns. Neuroimage 12, 314–325. 10.1006/nimg.2000.0612 10944414

[B5] Eisenberg N. Fabes R. A. Miller P. A. Fultz J. Shell R. Mathy R. M.. (1989). Relation of sympathy and personal distress to prosocial behavior: a multimethod study. J. Pers. Soc. Psychol. 57, 55–66. 2754604 10.1037//0022-3514.57.1.55

[B6] Fan Y. Duncan N. W. de Greck M. Northoff G. (2011). Is there a core neural network in empathy? an fMRI based quantitative meta-analysis. Neurosci. Biobehav. Rev. 35, 903–911. 10.1016/j.neubiorev.2010.10.009 20974173

[B7] Gusnard D. A. Akbudak E. Shulman G. L. Raichle M. E. (2001). Medial prefrontal cortex and self-referential mental activity: relation to a default mode of brain function. Proc. Natl. Acad. Sci. U.S.A. 98, 4259–4264. 10.1073/pnas.071043098 11259662 PMC31213

[B8] Hofmann S. G. Grossman P. Hinton D. E. (2011). Loving-kindness and compassion meditation: potential for psychological interventions. Clin. Psychol. Rev. 31, 1126–1132. 10.1016/j.cpr.2011.07.003 21840289 PMC3176989

[B9] Keltner D. Goetz J. L. (2007). Compassion, in Encyclopedia of Social Psychology, eds Baumeister R. F. Vohs K. D. (Thousand Oaks, CA: Sage Publications), 159–161.

[B10] Kim M. J. Gee D. G. Loucks R. A. Davis F. C. Whalen P. J. (2011). Anxiety dissociates dorsal and ventral medial prefrontal cortex functional connectivity with the amygdale at rest. Cereb. Cortex 21, 1667–1673. 10.1093/cercor/bhq237 21127016 PMC3116741

[B11] Klimecki O. M. Leiberg S. Ricard M. Singer T. (2014). Differential pattern of functional brain plasticity after compassion and empathy training. Soc. Cogn. Affect. Neurosci. 9, 873–879. 10.1093/scan/nst060 23576808 PMC4040103

[B12] Lamm C. Decety J. Singer T. (2011). Meta-analytic evidence for common and distinct neural networks associated with directly experienced pain and empathy for pain. Neuroimage 54, 2492–2502. 10.1016/j.neuroimage.2010.10.014 20946964

[B13] Lee T. M, Leung, M. K. Hou W. K. Tang J. C. Yin J. So K. F.. (2012). Distinct neural activity associated with focused-attention meditation and loving-kindness meditation. PLoS ONE 7:e40054. 10.1371/journal.pone.0040054 22905090 PMC3419705

[B14] Lehmann D. Faber P. L. Tei S. Pascual-Marqui R. D. Milz P. Kochi K.. (2012). Reduced functional connectivity between cortical sources in five meditation traditions detected with lagged coherence using EEG tomography. Neuroimage 60, 1574–1586. 10.1016/j.neuroimage.2012.01.042 22266174

[B15] Lutz A. Brefczynski-Lewis J. Johnstone T. Davidson R. J. (2008). Regulation of the neural circuitry of emotion by compassion meditation: effects of meditative expertise. PLoS ONE 3:e1897. 10.1371/journal.pone.0001897 18365029 PMC2267490

[B16] Pan W. Zhang L. Xia Y. (1994). The difference in EEG theta waves between concentrative and non-concentrative Qi-Gong states – a power spectrum and topographic mapping study. J. Tradit. Chin. Med. 14, 212–218. 7799657

[B17] Pardo J. V. Pardo P. J. Raichle M. E. (1993). Neural correlates of self-induced dysphoria. Am. J. Psychiatry 150, 713–719. 8480815 10.1176/ajp.150.5.713

[B18] Raichle M. E. MacLeod A. M. Snyder A. Z. Powers W. J. Gusnard D. A. Shulman G. L. (2001). A default mode of brain function. Proc. Natl. Acad. Sci. U.S.A. 98, 676–682. 10.1073/pnas.98.2.676 11209064 PMC14647

[B19] Reiman E. M. Lane R. D. Ahern G. L. Schwartz G. E. Davidson R. J. Friston K. J.. (1997). Neuroanatomical correlates of externally and internally generated human emotion. Am. J. Psychiatry 154, 918–925. 9210741 10.1176/ajp.154.7.918

[B20] Reva N. V. Pavlov S. V. Loktev K. V. Korenyok V. V. Aftanas L. I. (2014). Influence of long-term Sahaja Yoga meditation practice on emotional processing in the brain: an ERP study. Neuroscience 281, 195–201. 10.1016/j.neuroscience.2014.09.053 25281881

[B21] Shamay-Tsoory S. G. (2011). The neural bases for empathy. Neuroscientist 17, 18–24. 10.1177/1073858410379268 21071616

[B22] Shulman G. L. Fiez J. A. Corbetta M. Buckner R. L. Miezin F. M. Raichle M. E.. (1997). Common blood flow changes across visual tasks: II. decreases in cerebral cortex. J. Cogn. Neurosci. 9, 648–663. 23965122 10.1162/jocn.1997.9.5.648

[B23] Sonnetag S. Kuttler I. Fritz C. (2010). Job stressor, emotional exhaustion, and need for recovery: a multi-source study on the benefits of psychological detachment. J. Vocat. Behav. 76, 355–365. 10.1016/j.jvb.2009.06.005

[B24] Sperduti M. Martinelli P. Piolino P. (2012). A neurocognitive model of meditation based on activation likelihood estimation (ALE) meta-analysis. Conscious. Cogn. 21, 269–276. 10.1016/j.concog.2011.09.019 22005087

[B25] Tei S. Faber P. L. Lehmann D. Tsujiuchi T. Kumano H. Pascual-Marqui R. D.. (2009). Meditators and non-meditators: EEG source imaging during resting. Brain Topogr. 22, 158–165. 10.1007/s10548-009-0107-4 19653090

